# ECPUB5 Polyubiquitin Gene in *Euphorbia characias*: Molecular Characterization and Seasonal Expression Analysis

**DOI:** 10.3390/genes15070957

**Published:** 2024-07-21

**Authors:** Faustina Barbara Cannea, Daniela Diana, Rossano Rossino, Alessandra Padiglia

**Affiliations:** 1 Biomedical Section, Department of Life and Environmental Sciences (DiSVA), Cittadella Universitaria di Monserrato, University of Cagliari, 09042 Cagliari, Italy; faustinabarbara@tiscali.it; 2 Department of Medical Sciences and Public Health (DSMSP), AOU Presidio Microcitemico, University of Cagliari, 09121 Cagliari, Italy; dani.diana2@gmail.com (D.D.); rossino40@gmail.com (R.R.)

**Keywords:** *Euphorbia characias*, latex, polyubiquitin gene (ECPUB5), ubiquitin monomers, PCR-CODEHOP, molecular characterization, bioinformatics analysis, amino acid substitutions, drought stress

## Abstract

The spurge *Euphorbia characias* is known for its latex, which is rich in antioxidant enzymes and anti-phytopathogen molecules. In this study, we identified a novel polyubiquitin protein in the latex and leaves, leading to the first molecular characterization of its coding gene and expressed protein in *E. characias*. Using consensus-degenerate hybrid oligonucleotide primers (CODEHOP) and rapid amplification of cDNA ends (5′/3′-RACE), we reconstructed the entire open reading frame (ORF) and noncoding regions. Our analysis revealed that the polyubiquitin gene encodes five tandemly repeated sequences, each coding for a ubiquitin monomer with amino acid variations in four of the five repeats. In silico studies have suggested functional differences among monomers. Gene expression peaked during the summer, correlating with high temperatures and suggesting a role in heat stress response. Western blotting confirmed the presence of polyubiquitin in the latex and leaf tissues, indicating active ubiquitination processes. These findings enhance our understanding of polyubiquitin’s regulatory mechanisms and functions in *E. characias*, highlighting its unique structural and functional features.

## 1. Introduction

*Euphorbia characias* is a perennial shrub belonging to the *Euphorbiaceae* family, typically found in arid coastal environments with high salinity soils. A characteristic feature of *Euphorbiaceae* is the presence of laticiferous tissue synthesized by specialized cells containing latex, a milky sap with a complex composition [1,2]. Among the latex components, some metabolites, such as cardenolides (cardiokinetic glycosides) and a wide range of alkaloids, serve as important molecular reservoirs for plant defense against phytopathogens [3,4,5]. Previous studies have confirmed the presence of antioxidant enzymes in *E. characias* involved in the natural defense against abiotic and biotic stresses [6,7,8,9].

The molecular repertoire involved in defense against oxidative stress includes many proteins. Among these, ubiquitin deserves mention in addition to antioxidant enzymes. Ubiquitin plays a crucial role in the response of plants to drought stress, which is the most severe abiotic stress affecting plant growth and development. Drought stress induces a series of stresses, such as osmotic stress, oxidative stress, cellular damage, and alterations in cellular metabolism [10].

Ubiquitin is a small monomeric protein of 76 amino acids (8.5 kDa), whose name reflects its ubiquity, being present in all eukaryotic cells, where it serves as a molecular signal for protein degradation (ubiquitination). Ubiquitination involves the attachment of ubiquitin to a target protein through the activity of the E1 enzyme (ATP-dependent), followed by a second enzyme (E2), which allows the ubiquitin ligase (E3) to ubiquitinate the target proteins. The target proteins, which can be mono- or polyubiquitinated, resulting in different types of chains, are hydrolyzed by the proteasome [11,12,13]. Protein degradation by the ubiquitin–proteasome system is fundamental for controlling many physiological processes and counteracting various cellular stresses [14].

In eukaryotes, the response to heat shock involves the overexpression of genes coding for chaperones and proteins involved in ubiquitination aimed at eliminating misfolded proteins [15]. This response is crucial for maintaining protein homeostasis and cellular function under stress conditions. Chaperones assist in the proper folding of newly synthesized proteins and the refolding of misfolded proteins, thus preventing their aggregation. Simultaneously, the ubiquitination system tags irreparably damaged proteins for degradation by the proteasome, thus preventing the accumulation of potentially toxic protein aggregates [16,17]. This dual mechanism ensures that cells can rapidly adapt to environmental stress, maintaining protein quality control and cellular integrity. Studies have shown that the heat shock response is a highly conserved mechanism across different eukaryotic organisms, indicating its fundamental role in survival under stress conditions [18]. For instance, in *Arabidopsis thaliana*, heat shock proteins (HSPs) and ubiquitin-related pathways are upregulated in response to elevated temperatures, contributing to the plant’s ability to cope with heat stress [19,20]. Similarly, in mammals, the heat shock response not only helps in managing protein misfolding but also plays a role in modulating inflammation and apoptosis, further underscoring its importance in cellular defense mechanisms [21,22]. The ability of these genes to be highly expressed during periods of thermal stress suggests their role in enhancing the plant’s resilience by ensuring the rapid turnover of damaged proteins and maintaining cellular homeostasis [23]. This insight into the molecular mechanisms of stress responses can pave the way for developing crop varieties with improved stress tolerance, which is critical in the context of global climate change and increasing environmental stressors [24,25].

Ubiquitin plays a key role in multiple cellular functions, including protein quality control, cell proliferation regulation, DNA repair, and signal transduction mechanisms [26]. Numerous studies have demonstrated that even mild oxidative stress can promote ubiquitin expression [27,28,29,30,31], confirming that this highly conserved protein is directly involved in cellular homeostasis processes.

In eukaryotes, genes-encoding ubiquitin can be divided into two groups: the first group contains head-to-tail tandem repeats of 228 nucleotides, each coding for ubiquitin (poUbi; polyubiquitin genes) [32,33,34,35]; and the second group includes genes named Ub-CEP (ubiquitin carboxyl extension protein), which consist of a portion coding for a single N-terminal ubiquitin molecule joined with the genes coding for the large ribosomal protein UbL40 or the small ribosomal protein UbS27 [36,37,38]. Proteins produced by the poUbi and Ub-CEP genes are then processed by deubiquitinating enzymes that hydrolyze the peptide bonds to produce ubiquitin monomers or a ubiquitin monomer and one of the two ribosomal proteins, respectively.

In addition to poUbi and Ub-CEP genes, other genes coding for the different families of ubiquitin-like proteins named RUBs (related to ubiquitin proteins) have been described [39]. RUBs constitute a family of proteins with NH2-terminal domains that are significantly homologous to ubiquitin [40]. The analysis of over 1000 eukaryotic polyubiquitin coding sequences has highlighted considerable variability in the number of repeats resulting from duplication events [41]. The number of tandem repeats ranged from a minimum of 2 to a maximum of 14. It has been hypothesized that the optimal number of repeats is the result of evolutionary events that optimize the survival chances of species exposed to different types of stress. Transcripts with the tandem repeats of coding units represent a biomolecular strategy that allows cells to amplify protein biosynthesis processes and rapidly produce more molecules of a specific protein [41].

The primary structure of ubiquitin is identical across a wide range of organisms, from insects to phylogenetically distant organisms, such as yeasts, animals, and plants [42]. Ubiquitin expressed in yeast and humans differs by only three amino acid residues [32]. Ubiquitin has a secondary structure mainly consisting of five β-strands, two amphipathic α-helices, a short 310 helix, six β-reverse turns, and two β-hairpins [43]. Its tertiary structure is characterized by a core of 16–17 nonpolar amino acid residues, whose side chains interact via hydrophobic interactions. The structure is also stabilized by hydrogen bonds. The presence of this extended hydrophobic core and the hydrogen bonds confer structural stability to the protein, which explains its heat stability properties. When subjected to electrophoretic separation by SDS-PAGE, this 8.5 kDa protein migrates with an apparent molecular weight of 5.5 kDa [44], indicating its low tendency to completely lose its secondary and tertiary structure, even under the denaturation conditions required for SDS-PAGE separation.

Ubiquitin adopts a compact globular conformation to better bind covalently to target proteins. Ubiquitin residues Leu8, Val70, and Ile44 form a hydrophobic surface zone relevant to noncovalent interactions with many ubiquitin-binding domains (UBDs) [45,46,47]. Another fundamental residue for ubiquitin function is Gly-76 at the C-terminal end, which can bind covalently to one of the seven lysine residues (Lys6, Lys11, Lys27, Lys29, Lys33, Lys48, and Lys63) of another ubiquitin molecule. The binding between Gly76 and lysines can involve many ubiquitin molecules, resulting in the formation of ubiquitin chains [48,49].

The process of ubiquitination can be divided into monoubiquitination and polyubiquitination, depending on the number of bound ubiquitin molecules. Polyubiquitination, which involves a variable number of ubiquitin monomers, can be linear or branched depending on how the ubiquitin molecules are connected [50]. Monoubiquitination and polyubiquitination processes are mainly used to regulate the transcriptional activation of proteins and DNA repair systems [51]. Among the biological processes regulated by polyubiquitin chains, the most studied involve the Lys63 and Lys48 residues of monomers. In particular, polyubiquitin chains with Gly76–Lys63 linkages between monomers participate in DNA repair processes [52]. Monomers linked with Gly76–Lys48 bonds to form polyubiquitin chains are involved not only in DNA damage repair but also in regulating protein stability [53]. Numerous studies have also highlighted the role of Lys11 in forming polyubiquitin chains that mediate cell cycle regulation [54,55] and the role of Lys33 in intracellular protein transport and cytophagy [56]. The importance of ubiquitination in plant growth and development has been confirmed by numerous studies on *A. thaliana* [57,58].

The primary objective of this study was to characterize the polyubiquitin gene in *E. characias*. Specifically, we aimed to (1) elucidate the structure of the polyubiquitin gene; and (2) analyze its seasonal expression with a focus on the warmer months to determine the expression patterns related to seasonal and climatic variations; and (3) identify and localize the polyubiquitin protein in latex and leaf tissue. Additionally, we aimed to provide data and analyses to enhance our understanding of the regulation and function of polyubiquitins in plants, particularly within the *Euphorbiaceae* family. The molecular insights gained from this work could be correlated with regulation and function in future studies, paving the way for advancements in agronomy and biotechnology research.

## 2. Materials and Methods

### 2.1. Plant Material

**Figure 1:**
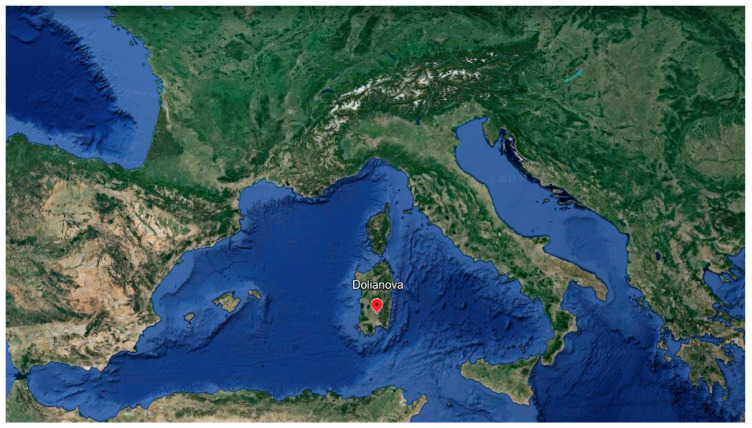
This satellite view was obtained through Google Earth of the Mediterranean Sea, where Sardinia occupies a central position. The location of Dolianova is indicated with a red point.

The latex and young leaves of *E. characias* were collected from January to December in Dolianova in southern Sardinia, Italy (latitude 39°32′04″ N, longitude 9°06′24″ E) (Figure 1). The samples were frozen and stored at −20 °C until use. All chemicals were obtained as pure commercial products and used without further purification.

### 2.2. Isolation of RNA and cDNA Synthesis

Total RNA was extracted from *E. characias* latex, as previously described [59]. For RNA extraction from latex, *E. characias* branches were cut, and the fresh latex (~5 mL) was collected directly into a tube containing 20 mL of 2 × RNA extraction buffer (0.1 M Tris-HCl, 0.3 M LiCl, 0.01 M EDTA, 10% SDS, pH 9.5) along with 5 mL of RNAlater solution (Sigma-Aldrich, St. Louis, MO, USA) to stabilize and protect the RNA. The latex solution was mixed and centrifuged at 8000× *g* for 15 min at 20 °C. The supernatant was processed using TRI Reagent (Sigma-Aldrich) according to the manufacturer’s instructions.

For RNA extraction from leaves, RNA was isolated from 0.5 g of liquid nitrogen-frozen young leaves of *E. characias* powder using the RNAqueous isolation kit (Ambion, Austin, TX, USA) and the Plant Isolation Aid reagent (Ambion) to increase the RNA yield, following the manufacturer’s instructions. The quality of purified RNA was verified by gel electrophoresis using a 1% denaturing agarose gel stained with SYBR Green II (Sigma-Aldrich), and concentrations were measured using a NanoDrop 2000c UV–VIS Spectrophotometer (Thermo Scientific, Waltham, MA, USA) at 260 nm.

To obtain cDNA, RNA from *E. characias* latex and leaves was reverse transcribed with an oligo(dT) primer using an enhanced avian myeloblastosis virus reverse transcriptase enzyme (Sigma-Aldrich), following the manufacturer’s instructions. All PCR and nucleic acid blotting experiments described below were carried out using both young leaves and latex of *E. characias* cDNA. These methods were chosen to ensure the accurate and reliable quantification of gene expression under varying environmental conditions, particularly focusing on stress responses related to heat and drought.

### 2.3. Amplification of E. characias cDNAs by PCR with Hybrid Primers

To identify the unknown nucleotide sequences of the polyubiquitin gene, we employed a degenerate hybrid oligonucleotide primer (CODEHOP) strategy [60]. This process begins by aligning multiple sequences of polyubiquitin proteins. Five different plant sources were chosen from the GenBank SwissProt database: *A. thaliana* (Accession No. Q9SAQ6), *Solanum lycopersicum* (Accession No. Q40164), *Pisum sativum* (Accession No. Q41754), *Zea mays* (Accession No. Q01867), and *Olea europaea* (Accession No. Q9SNZ1). These sequences were aligned using Clustal Omega (https://www.ebi.ac.uk/Tools/msa/clustalo/, accessed on 9 June 2024) (Figure S1) and then cut into blocks using Block Marker software [61]. Primers were designed using the default parameters of the j-CODEHOP server (https://4virology.net/virology-ca-tools/j-codehop/, accessed on 9 June 2024).

Amplification primers for *E. characias* polyubiquitin cDNA were selected from a group of primer candidates provided by the j-CODEHOP program. Each primer included the consensus clamp in the uppercase and the degenerate core in the lowercase: y = [CT]; r = [AG]; n = [AGCT]. PCR was performed in a solution containing 1.5 mM MgCl2, 100 mM Tris–HCl (pH 8.3), 50 mM KCl, 200 mM dNTP mix, 1 μM sense primer, 1 μM antisense primer, 1 μg of *E. characias* cDNA, and 1–3 units of JumpStart AccuTaq LA DNA polymerase mix (Sigma-Aldrich). Thermal cycles of amplification were carried out in a Personal Eppendorf Mastercycler (Eppendorf, Hamburg, Germany) using slightly different programs.

Thermal cycles of amplification were carried out in a Personal Eppendorf Mastercycler (Eppendorf, Hamburg, Germany) using slightly different programs. Specifically, fragments of approximately 1150 bp, 900 bp, 690 bp, 460 bp, and 200 bp were obtained using the sense primer F1 (5′-ATGCAGATCttygtnaarac-3′) with the antisense primer R1 (5′-GGTCAGGGTCTTCACGAAGatytgcatncc-3′) and the sense primer F2 (5′-TGAAGGCCAAGATCCAGgayaargargg-3′) with the antisense primer R2 (5′-TGGTCAGGAGGGATGccytcyttrtc-3′). The PCR conditions were as follows: initial denaturation at 94 °C for 3 min; followed by 35 cycles of denaturation at 94 °C for 30 s, annealing at 50 °C for 45 s, an extension at 72 °C for 30 s, and a final extension at 72 °C for 7 min. The position and orientation of these primers relative to the gene are shown in Figure S1. The PCR products detected on 6% polyacrylamide or 2% agarose gels were purified using a ChargeSwitch PCR Clean-Up kit (Invitrogen, Carlsbad, CA, USA) and then sent to BioFab (Rome, Italy) for sequencing. The nucleotide sequences were translated into amino acid sequences in silico using the ExPASy translate tool (https://web.expasy.org/translate/, accessed on 9 June 2024). Sequences were aligned using Clustal Omega, and similarities were analyzed with the advanced BLAST algorithm available on the National Center for Biotechnology Information website (http://www.ncbi.nlm.nih.gov/, accessed on 9 June 2024) and the FASTA algorithm v.3.0 from the European Bioinformatics Institute website (http://www.ebi.ac.uk/fasta33/index.html, accessed on 9 June 2024).

### 2.4. cDNA Blotting Analysis

We used the cDNA blotting technique for two main purposes: first, to determine the size of the entire transcript detected with the CODEHOP primers, enabling us to perform gene mapping experiments to identify the complete gene sequence, and second, to check for the presence of one or more ubiquitin transcripts in *E. characias* latex and leaves, which could result from alternative splicing or other polyubiquitin-coding genes.

**Figure 2:**
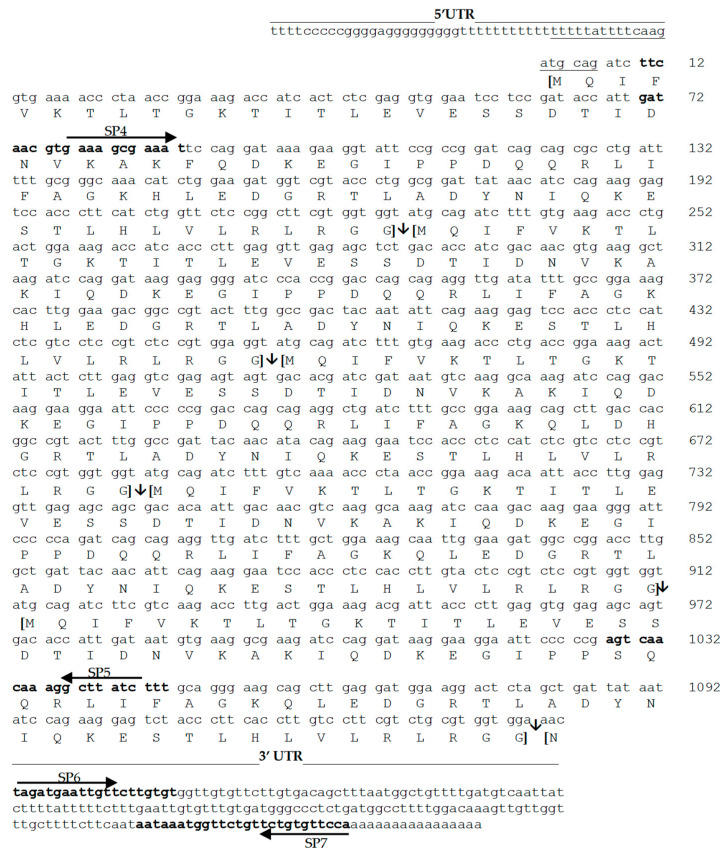
Nucleotide and amino acid sequence of *E. characias* polyubiquitin (ECPUB5). The ORF (1143 bp) consists of 5 tandem repeats, represented in parentheses. The 3′-UTR (48 bp) and 5′-UTR (191 bp) are indicated by a solid line above the block of nucleotides. Arrows (↓) indicate cleavage sites caused by deubiquitinase enzymes. The 3′-UTR includes a putative polyadenylation signal “aataaa” (in bold underlined), located 25 bp upstream of the poly(A) tail. The position and orientation of the specific sense (SP4, SP6) and antisense (SP5, SP7) primers relative to the gene are shown with arrows above or below the bold sequence. The numbering refers to the nucleotide coding sequence.

The cDNAs synthesized from total RNA (5 µg) were electrophoresed on a 1% agarose gel and transferred overnight to a positively charged nylon membrane (Roche Diagnostics, Mannheim, Germany) by capillary blotting in 20× saline–sodium citrate (SSC) buffer (3 M NaCl, 0.3 M trisodium citrate, pH 7.0). The blot was baked for 30 min at 120 °C. The PCR fragment to be used as a probe was obtained with a pair of specific primers designed from the sequence of the 1150 bp product obtained by CODEHOP PCR. The sense primer SP4 (5′-GAT AAC GTG AAA GCG AAA-3′), designed from the sequence of the DNVKAKF peptide together with the antisense primer SP5 (5′-AAAGATAAGCCTTTGTTGACT-3′), designed from the sequence of the SQQRLIF peptide, allowed us to obtain a single PCR product of 1098 bp. The position and orientation of these primers relative to the gene are shown in Figure 2.

The fragment obtained was labeled with digoxigenin-dUTP, as described in the DIG System User’s Guide (Roche Diagnostics). Hybridization was performed overnight at 42 °C in Ultrahyb hybridization solution (Ambion) at a probe concentration of 5 ng/mL. The membrane was washed twice in 2 × SSC, 0.1% SDS for 5 min, and twice in 0.1 × SSC, 0.1% SDS for 15 min at 42 °C. The DIG-labeled hybridized cDNA was detected with an enzyme-linked immunoassay using an anti-DIG antibody conjugated to alkaline phosphatase. The chromogenic substrate 5-bromo-4-chloro-3-indolyl phosphate (BCIP)/nitro blue tetrazolium (NBT) was then used to detect the DIG-labeled probe. A 0.13–23.1 kb DNA ladder was used as the size marker (Roche Diagnostics).

### 2.5. Rapid Amplification of cDNA Ends (RACE)

Rapid amplification of the 5′ and 3′ ends was performed as described by Frohman [62], using antisense-specific primers and the anchor primer provided in the RACE kit (Roche Diagnostics). For 5′ RACE, the antisense-specific primer SP1 (5′-TCTGGTTCTCCGGCTTCGTGGTGGTA-3′) was used in a reverse transcription reaction with 2 µg *E. characias* total RNA. First-strand cDNA was purified from unincorporated nucleotides and primers using the High-Pure PCR Purification Kit (Roche Diagnostics). A homopolymeric tail was added to the 3′ end of the RT-PCR products, and the cDNA was amplified by PCR using the nested antisense-specific primer SP2 (5′-ACCATCTTCCAGATGTTTGCCCGCAAA-3′) and the oligo(dT)-anchor primer provided in the RACE kit, following the supplied protocol. For the 3′ RACE, the first-strand cDNA was obtained using the oligo(dT)-anchor primer and amplified using the sense-specific primer SP3 (5′-GACACCATTGATAATGTGAAGGCGAAG-3′) and the antisense PCR anchor primer provided in the RACE kit. PCR reactions were performed using 1–3 units of JumpStart AccuTaq LA DNA polymerase mix (Sigma-Aldrich) under different experimental conditions. The PCR products obtained from the RACE experiments were sent for sequencing (BioFab, Rome, Italy). The position and orientation of the sense- and antisense-specific primers relative to the gene are shown in Figure 2.

### 2.6. In Silico Reconstruction of the Full-Length Sequence

To reconstruct the full-length sequence of the *E. characias* polyubiquitin gene, we combined the data obtained from the CODEHOP PCR and RACE methodologies. The sequences generated from the CODEHOP PCR provided the initial fragments, while the RACE method allowed for the extension of the 5′ and 3′ ends of the gene.

Initially, the sequences obtained from the CODEHOP PCR were aligned and assembled using Clustal Omega. These fragments provided partial sequences, which were then extended using the sequences obtained from 5′ and 3′ RACE. The 5′ RACE products were aligned with the 5′ end of the CODEHOP PCR fragments, and the 3′ RACE products were aligned with the 3′ end of the CODEHOP PCR fragments. This alignment and assembly process was performed using the software Geneious 2024 (https://www.geneious.com/).

The final full-length sequence was then validated by comparing it against the reference sequences available in the NCBI database using BLAST. After reconstruction, the gene was designated with the acronym ECPUB5 (indicating a polyubiquitin gene consisting of five tandem repeats), and the corresponding ubiquitin proteins derived from this gene were designated with the acronym ECUB. The resulting full-length sequence (Figure 2) of ECPUB5 was submitted to the NCBI nucleotide database with the accession number DQ011576, and the corresponding protein sequence was submitted to the NCBI protein database with the accession number AAY33920.2.

### 2.7. Gene Expression Analysis of ECPUB5

To evaluate the expression of the ECPUB5 gene in *E. characias* latex and leaf tissue, we employed both Northern blot and quantitative RT-PCR (qRT-PCR) methods. The experiments described below were conducted at approximately bimonthly intervals. Specifically, samples were collected in February, April, June, August, October, and December to capture the significant climatic fluctuations occurring every 2 months. This timeline was chosen to observe seasonal variations in temperature and humidity, allowing us to correlate mRNA expression with environmental changes.

#### 2.7.1. Northern Blot Analysis

We used the Northern blot method to test whether the ECPUB5 gene expression underwent seasonal fluctuations. Total RNA (10 µg) was obtained from latex and leaf samples collected bimonthly from February to December of the same year. These samples were frozen and stored at –80 °C until use. For sample preparation, total RNA (10 µg) was dried in a Speedvac, dissolved in 20 µL of RNA sample loading buffer (Sigma-Aldrich), and heated to 60 °C for 10 min. The samples were then snap-cooled on ice and loaded onto a 1.2% agarose gel with 1 × MOPS (0.2 M MOPS, 10 mM EDTA, 0.5 M sodium acetate, pH 7) containing 0.66 M formaldehyde and ethidium bromide. A 0.28–6.5 kilobase RNA marker (Roche Diagnostics) was also loaded. The gel was run at 5 V/cm in 1 × MOPS buffer, and then the RNA was transferred overnight to a positively charged nylon membrane (Roche Diagnostics) by capillary blotting in 20 × SSC buffer (3 M NaCl, 0.3 M trisodium citrate, pH 7.0). The blot was baked for 30 min at 120 °C. Hybridization conditions, probe labeling, and filter washing steps were identical to those described in the Southern blot analysis section.

#### 2.7.2. Quantification of ECPUB5 Expression in *E. characias* Leaves and Latex Using Quantitative RT-PCR (qRT-PCR)

Quantification of ECPUB5 gene expression was also evaluated by absolute qRT-PCR. The standard curve was obtained from the known concentrations of ECPUB5 cDNA. To ensure a homogeneous concentration of cDNA molecules, given the nucleotide redundancy present in the gene, we used the sense primer SP4, which only hybridizes in the first of the five tandem repeats, and the antisense primer SP7, which binds to the 3′ UTR (Figure 2). The cDNA obtained was then purified using a Thermo Fisher Scientific GeneJET PCR Purification Kit (Thermo Fisher Scientific, Waltham, MA, USA). The concentration of the purified cDNA was measured using NanoDrop, and the cDNA copy number was calculated using the Science Primer tool (https://scienceprimer.com/). The cDNA was stored at −80 °C until use. To generate the standard curve, scalar concentrations of cDNA were used, starting from an initial concentration of 100 ng/µL.

qRT-PCR was performed using SYBR Green technology on the StepOne Real-Time PCR System (Applied Biosystems, Foster City, CA, USA). The sense primer SP6 (5′-TAGATGAATTGTTCTTGTGT-3′) and the antisense primer SP7 (5′-TGGAACACAGAACCA-3′) were designed on the 3′-UTR region of ECPUB5, which is a unique and non-repeated sequence (Figure 2). Amplifications were carried out in a 96-well plate in a 25 μL reaction volume containing 12.5 μL of 2 × SYBR Green Master Mix (Applied Biosystems), 2.5 μL of each sense and antisense primer (10 mM), 1 μL of template (1 µg ECPUB5 cDNA or different concentrations of standard ECPUB5 cDNA), and 9 μL of DEPC water. The thermal profile for qRT-PCR with SYBR Green was 50 °C for 2 min and 95 °C for 10 min, followed by 40 cycles at 95 °C for 15 s and 60 °C for 1 min. Each sample was run in triplicate in a 96-well plate with DEPC water used as a negative control instead of the template.

Once the analysis was finished, linear relationships between the Ct (PCR threshold cycle) and different logarithmic concentrations of the ECPUB5 standard were automatically generated using StepOne Real-Time PCR V2.0 software (Applied Biosystems). The different Ct values of ECPUB5 were calculated as the effective concentrations of ECPUB5 mRNA in total RNA based on the standard curve obtained with the various concentrations of the ECPUB5 standard used (R^2^ = 0.999).

### 2.8. Primer Sequences

All primer sequences used in this work are displayed in Table S1.

### 2.9. Total Protein Quantification

Total proteins were detected in both the latex and leaf tissues of *E. characias*. The samples were sonicated in RIPA buffer (50 mM Tris–HCl, pH 8.0, 150 mM sodium chloride, 1.0% Igepal CA-630 (NP-40), 0.5% sodium deoxycholate, and 0.1% sodium dodecyl sulfate), supplemented with a protease inhibitor (Bio-Rad Laboratories Inc., Hercules, CA, USA). The samples consisted of 25 mg of leaf tissue and 50 µL of latex per 100 µL of buffer. The tubes were centrifuged at 14,000× *g* for 20 min to obtain the supernatant. The amount of total protein was determined using the Pierce BCA Protein Assay Kit (Thermo Fisher Scientific), with bovine serum albumin (BSA) as a standard. A standard curve was created by serial dilution of BSA in RIPA buffer. The assay for each sample was carried out according to the manufacturer’s instructions. Triplicates were run for each sample and standard. The absorbance was measured at 562 nm using a Bio-Rad 680 microplate reader.

### 2.10. SDS-PAGE and Western Blot Analysis

We chose to perform the Western blot analyses on samples collected in August, during the period of highest gene expression, to gain more information on the presence of the ECUB protein.

For the analyses, we used a Mini-PROTEAN electrophoresis system (Bio-Rad Laboratories Inc.) with a 10% Mini-PROTEAN TGX precast gel to separate the proteins from the latex and leaf tissue samples. SDS–PAGE was performed using a prestained Perfect Color Protein Ladder (EURx Ltd., Gdańsk, Poland) as a protein marker. For sample preparation, 5 µL of the supernatant from each fraction (containing 25 µg of protein) was mixed with 2 × Laemmli sample buffer (Bio-Rad Laboratories Inc.) under reducing and non-reducing conditions and heated at 95 °C for 5 min before loading onto the gel.

For Western blot analysis, gel-loading homogenates were prepared, as previously described. Proteins from SDS–PAGE were transferred onto a 0.2 μm nitrocellulose membrane (Bio-Rad Laboratories Inc.), which had been previously immersed in transfer solution prepared from Bio-Rad 5 × transfer buffer, using the Trans-Blot Turbo Transfer System (Bio-Rad Laboratories Inc.). After protein transfer, the membrane was rinsed with TBST (100 mM Tris-HCl, 150 mM NaCl, 0.05% [*v*/*v*] Tween 20, pH 7.5) and then blocked in blocking buffer (TBST containing 5% milk powder) for 45 min at room temperature. The membrane was subsequently incubated with the anti-ubiquitin antibody (Ubiquitin (P37) Antibody, Euroclone) at a 1:500 dilution for 1 h. After three washes in TBST, the membrane was incubated for 1 h at room temperature with a peroxidase-conjugated rabbit anti-IgG secondary antibody at a 1:20,000 dilution. Finally, the membrane was washed with TBST and developed using tetramethylbenzidine (TMB) peroxidase chromogenic substrate (Sigma-Aldrich), following the manufacturer’s instructions.

### 2.11. Secondary Structure Prediction

The amino acid sequences coding for the five ubiquitin monomers were subjected to secondary protein structure prediction using the Statistical Analysis of Biological Labelled Entities (SABLE) tool [62,63,64], available at https://sable.cchmc.org/ (accessed on 15 February 2024). The SABLE tool was selected due to its effectiveness in predicting alpha helices and beta sheets based on the propensities of individual amino acids. The prediction tool utilizes neural networks and statistical algorithms to analyze sequence data and predict secondary structure elements with high accuracy.

For the analysis, the following steps were undertaken:

Sequence preparation: The amino acid sequences of the ubiquitin monomers were obtained from the translated nucleotide sequences of the ECPUB5 gene and submitted to the SABLE prediction server.

Prediction parameters: The tool parameters were set to default values, which included the use of a sliding window approach for averaging propensities over multiple residues.

Prediction and visualization: SABLE generated predictions for alpha helices, beta sheets, and coils. The predicted secondary structures were visualized and analyzed to identify folding patterns and potential functional domains.

Comparison with experimental data: The predicted secondary structures were compared with available experimental data, such as those from X-ray crystallography and NMR, obtained from the Protein Data Bank (PDB), to validate the computational results. Discrepancies between the predicted and experimental structures were noted and investigated.

Additionally, BLAST (basic local alignment search tool) was used for sequence alignment and homology comparison to identify the conserved structural motifs critical for the protein’s biological function. These comprehensive analyses provided insights into the folding patterns and potential functional domains of ubiquitin monomers. This detailed methodology ensures the reproducibility and validation of the predicted functional differences among the monomers, enhancing the robustness of our findings.

### 2.12. Availability of Sequence Data

The nucleotide sequence characterized in this study was deposited in the NCBI nucleotide database with the accession number DQ011576. The corresponding protein sequence is available in the NCBI protein database with the accession number AAY33920.2.

## 3. Results

The sequencing of ECPUB5 cDNA established that *E. characias* ubiquitin (ECUB) is transcribed from a gene consisting of five tandem repeats, each encoding a ubiquitin monomer with different characteristics at the primary structure level. The expression of ECPUB5 RNA during the different months of the year was also assessed, and the ECUB protein was detected in both free form and bound to the target proteins. The results of this study, carried out on the latex and leaves, confirmed the expression of the ECPUB5 gene in both the latex and leaves of the plant. The data obtained were subjected to bioinformatic analysis to understand the structure and characteristics of the ECPUB5 gene, confirming its homology with the known polyubiquitin genes of other plant and animal species. The experimental data reported in this work not only demonstrated the presence of a gene encoding for a polyubiquitin but also contribute to the genetic knowledge of this plant and enrich genetic databases with new nucleotide sequences.

### 3.1. Cloning and Analysis of Polyubiquitin cDNA

Using the J-CODEHOP program, two pairs of forward and reverse primers were designed, allowing us to obtain fragments in the 1150–200 bp range in the PCR experiments. Specifically, with the available pairs of primers, we simultaneously amplified the tandemly repeated sequences of the ECPUB5 gene. Sequencing the fragments obtained with the CODEHOP technique allowed us to reconstruct approximately 80% of the entire ECPUB5 gene sequence and proceed with the design of the sense- and antisense-specific primers to be used in the RACE experiments. By pooling the results obtained after sequencing the cDNA fragments obtained by PCR-CODEHOP and RACE, we obtained the entire coding sequence and the untranslated UTR regions. By elaborating on the results obtained during the PCR-CODEHOP experiments and 5′/3′-RACE, we hypothesized the presence of an ECPUB5 gene of 1382 bp in *E. characias*, including the 5′ and 3′ UTR regions of 48 bp and 191 bp, respectively (Figure 2). The primary structure deduced in silico highlighted the presence of five tandemly repeated amino acid sequences, each corresponding to a ubiquitin monomer.

### 3.2. Sequence Alignment and Analysis

Alignment of the ECPUB5 sequence with those of other *Euphorbiaceae* species revealed significant differences in repeats 1, 2, 3, and 5. Specifically, compared to other polyubiquitins, we observed the I30F and Q49H substitutions in the first block, the Q125H substitution in the second block, the E203D and D204H substitutions in the third block, and finally, the D243S substitution in the fifth block (Figure S2). These variations were not observed in the primary structure of the other polyubiquitins deposited in the database.

### 3.3. Primary Structure of ECPUB5 Sequence: In Silico-Deduced Analysis

**Figure 3:**
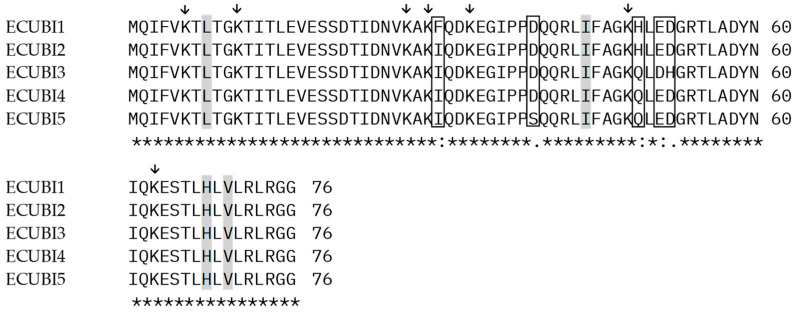
Amino acid alignment of each repetition (ECUBI 1-5) of ECPUB5. The conserved amino acid residues forming the Ile-44 patch core (Leu 8, Ile 44, His 68, Val 70) are highlighted in gray. Lysine residues important for the formation of polyubiquitin chains are indicated with arrows (↓). Non-identical amino acids are shown in boxes. Asterisks (*) under the aligned sequences indicate fully conserved residues across all repetitions.

To verify any differences in the primary structure, each repeating sequence was aligned with the others using the Clustal Omega tool (version 1.2.4, https://www.ebi.ac.uk/jdispatcher/msa/clustalo, accessed on 9 June 2024), showing the same differences. This is unusual, since the alignment gave an identity equal to 100% in the case of the polyubiquitin monomers reported in the database. However, we highlighted that the amino acids fundamental to the activity of the protein are conserved (Figure 3). In particular, the Ile44, Leu8, Val70, and His68 residues, which form the Ile-44 patch core for interaction with the proteasome [46,47], are present in each of the five repetitions. Other conserved amino acids are Gly76 and the seven lysine residues (Lys6, Lys11, Lys27, Lys29, Lys33, Lys48, and Lys63), which are important in the formation of polyubiquitin chains (Figure 3).

### 3.4. Secondary Structure of ECUB Monomers: In Silico-Deduced Analysis

**Figure 4:**
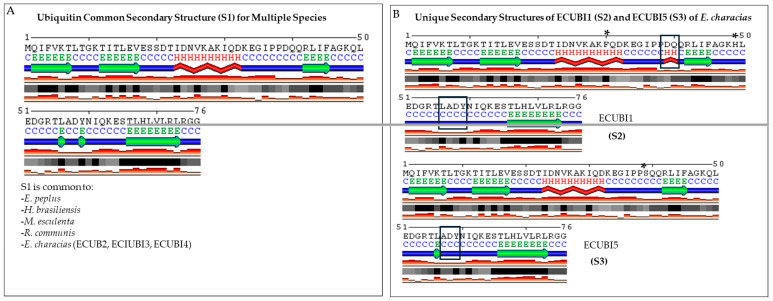
Comparison of the secondary structure predictions of ubiquitin monomers derived from polyubiquitin genes with five tandem repeats in *Euphorbiaceae* species. Panel (**A**) shows the common secondary structure (S1) shared by *Euphorbia peplus*, *HHevea brasiliensis*, *Morchella esculenta*, *Ricinus communis*, and monomers 2, 3, and 4 of *E. characias*. Panel (**B**) shows the unique secondary structures (S2 and S3) of monomers 1 and 5 of *E. characias*, respectively. The letter “S” stands for the secondary structure. The differences are highlighted in boxes. The asterisks (*) above the ECUBI1 and ECUBI5 sequences indicate amino acid residues that are not present in the other ubiquitins. These differences led to the in silico prediction of the S2 and S3 structures. The S2 region of ECUBI1 and the S3 region of ECUBI5, which have different secondary structures compared to S1, are shown in boxes. The letters used to identify the particular types of secondary structure are C (coil), E (extended strand), H (helix), and T (turn).

To determine whether the deduced amino acid substitutions could give rise to different secondary structures, we subjected the five ECUB monomer amino acid sequences (ECUB1–5) to bioinformatics analysis using the SABLE structural prediction tool. The in silico reconstruction of the secondary structures showed differences in the distribution of the coils and extended strands in the ECUB1 and ECUB5 monomers compared to the other three ECUBI monomers and to the monomers derived from other polyubiquitin proteins. Specifically, the F30 and H49 residues of the ECUB1 monomer, which replace the I30 and Q49 residues present in the other monomers, respectively, cause the disappearance of a coil and an extended strand, and the formation of a helix and a coil in the same positions. Moreover, the S39 residue of the ECUB5 monomer, which replaces the D39 residue present in the other monomers, causes the disappearance of an extended strand and the formation of a coil in the same position. The other substitutions in the ECUB2, ECUB3, and ECUB4 monomers, however, did not involve structural modifications (Figure 4).

### 3.5. Northern Blot Analysis and qRT-PCR

**Figure 5:**
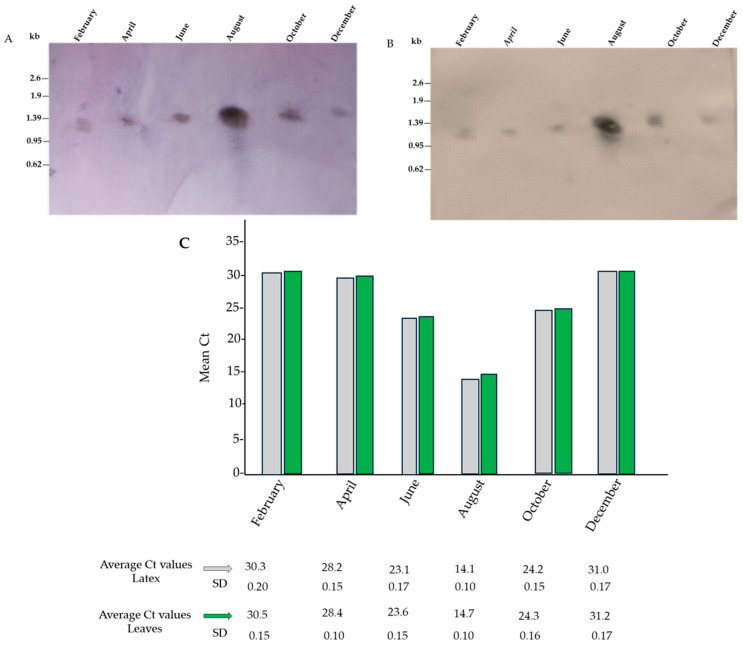
Northern blot and qRT-PCR analyses of the expression of mRNA encoding ECPUB5 over the course of the year (bimonthly analysis from February to December). (**A**) Northern blot analyses performed on RNA extracted from latex and (**B**) on RNA extracted from leaves. (**C**) ECPUB5 expression is presented as the threshold PCR cycle (Ct) mean value in the different samples. The average Ct values of the three replicates and the standard deviation (SD) are reported. The results reveal a prominent band in the RNA samples collected in August, which is the hottest and driest period of the year, with average temperatures reaching approximately 32 °C.

Total RNAs from *E. characias* latex and leaves were subjected to Northern blotting using DIG-labeled *E. characias* ECPUB5 cDNA as a hybridization probe to investigate the expression of ECPUB5 mRNA. The experiments showed a single ECPUB5 hybridization band of about 1.4 kb. This finding revealed that a homogeneous ECPUB5 mRNA population occurred in *E. characias* and that alternative splicing events or the expression of other genes coding for ubiquitin did not occur. Northern blotting experiments also showed that the gene expression of ECPUB5 in latex and leaves underwent seasonal fluctuations. Intensive hybridization bands were detectable in the RNA samples isolated and analyzed in August, whereas weak signals were detectable in samples isolated and examined during the colder months (Figure 5A,B). To confirm this result, the total RNA was isolated from latex and leaves at approximately monthly intervals from January to December, and reverse transcribed to cDNAs to perform qRT-PCR. The expression analysis of ECPUB5 revealed significant upregulation during the hottest and driest months, with mRNA levels being approximately 32-fold higher in August than in February (Figure 5C). This indicates a strong correlation between the ECPUB5 expression and environmental stress conditions. Indeed, in August, the average temperatures exceeded 32 °C, and severe drought conditions were recorded.

### 3.6. ECUB Protein Detection

**Figure 6:**
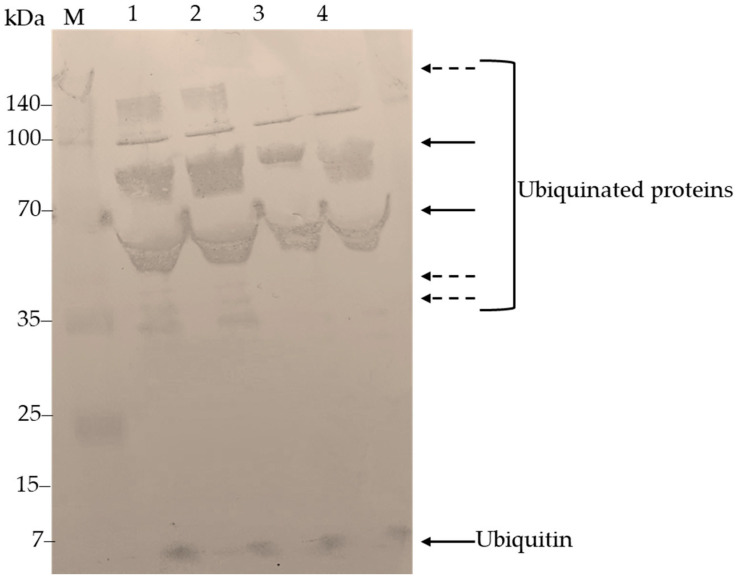
Western blot analysis results. Lanes 1 and 2 contain samples from latex and leaf tissue, respectively, under reducing conditions. Lanes 3 and 4 contain samples from latex and leaf tissue, respectively, under non-reducing conditions. The samples were collected in August. Solid arrows indicate an immunoreactive band at approximately 5–6 kDa, likely corresponding to the ubiquitin monomer, as well as several immunoreactive bands above 50 kDa, likely representing ubiquitinated proteins. Dashed arrows highlight immunoreactive bands of about 35 kDa present only in samples prepared under reducing conditions, suggesting that these bands correspond to protein complexes or aggregates that dissociate in the presence of reducing agents.

As a final step in this work, we used Western blotting with an anti-ubiquitin antibody to detect the protein in *E. characias*. Specifically, we used latex and leaf tissue samples collected during the hottest and driest periods of the summer season. The antibodies identified two main proteins of approximately 5–6 kDa and several immuno-reactive bands above 50 kDa, likely representing ubiquitinated proteins (Figure 6). According to the literature, when subjected to electrophoretic separation by SDS-PAGE, ubiquitin with a molecular weight (MW) of 8.5 kDa migrates with an apparent molecular weight of 5.5 kDa [44], indicating the ubiquitin’s low tendency to lose its secondary and tertiary structure during denaturation treatments in SDS-PAGE. Thus, the lower molecular weight band could be related to the presence of the ubiquitin monomer. Conversely, bands above 50 kDa could be related to the presence of ubiquitinated proteins. Similarly, higher molecular weight bands could correspond to ubiquitinated proteins. Samples prepared under reducing conditions indicate that these bands correspond to protein complexes or aggregates that dissociate in the presence of reducing agents.

## 4. Discussion

This paper reports the results of the molecular characterization of a polyubiquitin gene present in *E. characias* (ECPUB5), a plant widespread in various arid areas of Sardinia, and widely studied for the peculiarity of its latex, which is particularly rich in protein and non-protein molecules important for providing the plant with an effective defense system against biotic and abiotic stresses.

This study provides a comprehensive molecular characterization of the ECPUB5 gene in *E. characias*, highlighting several key findings. The ECPUB5 gene consists of five tandem repeats, each encoding a ubiquitin monomer with unique variations in the primary structure. These variations, specifically the I30F, Q49H, Q125H, E203D, D204H, and D243S substitutions, distinguish the ECPUB5 gene from other known polyubiquitins in *Euphorbiaceae*.

### 4.1. Unique Amino Acid Variations

The in silico analysis revealed that, despite these variations, the amino acids critical for ubiquitin function, such as Ile44, Leu8, Val70, His68, Gly76, and the seven lysine residues, are conserved. This suggests that the essential biological activities of ubiquitin, including its role in protein degradation via the proteasome, are preserved in ECPUB5. Unique variations in the primary structure, such as I30F, Q49H, and D39S substitutions, may enhance protein stability or interaction under stress conditions. For instance, Lyzenga and Stone [65] demonstrated that specific amino acid changes in ubiquitin can influence stress tolerance in plants, while Vierstra [66] highlighted the role of ubiquitin in maintaining protein homeostasis under stress. Our results support these findings, indicating that ECPUB5 might confer adaptive advantages to *E. characias* in response to environmental stress.

### 4.2. Structural Insights

Secondary structure predictions indicated a unique difference in the ECUB1 and ECUB5 monomers. In the ECUB1 monomer, the substitution of residues I30 and Q49 with F30 and H49 resulted in the loss of a coil and an extended strand, and the subsequent formation of a helix and a coil. In the ECUB5 monomer, the replacement of D39 with S39 led to the disappearance of an extended strand and the creation of a coil at the same site. Similar structural changes have been observed in the polyubiquitin genes of other species, influencing protein stability and function [67]. These structural alterations could affect the protein’s interaction with other molecules, potentially enhancing the plant’s ability to cope with stress. Pickart and Eddins [24] discussed how structural variations in ubiquitin can affect its binding affinity and interaction with other proteins, which could be particularly relevant for the adaptive mechanisms of *E. characias*.

### 4.3. Seasonal Expression Patterns

Seasonal expression analysis showed that ECPUB5 mRNA levels and ECUB protein concentrations were significantly higher during the hottest and driest months, particularly in August. This increase, confirmed by both Northern blot and qRT-PCR, suggests that ECPUB5 plays a critical role in the plant’s response to thermal and drought stress. The upregulation of polyubiquitin genes under stress conditions is consistent with their known functions in protein homeostasis and stress responses [68,69]. Our findings are supported by previous studies, such as those by Glickman and Ciechanover [53] and Xu et al. [70], which demonstrated the crucial role of ubiquitin in plant stress responses. The significant increase in ECPUB5 expression during the hot summer months indicates a potential role in the plant’s defense mechanisms against environmental stressors. This correlation between high temperatures, drought conditions, and an elevated ECPUB5 expression suggests that this gene is part of the plant’s adaptive response to extreme conditions, helping to maintain protein integrity and function.

### 4.4. Protein Detection and Implications

The antibodies used in the Western blot analysis detected proteins with an approximate molecular mass of 5–6 kDa, as well as multiple immunoreactive bands exceeding 50 kDa, likely indicative of ubiquitinated proteins. The presence of ubiquitinated proteins highlights the involvement of ECPUB5 in the ubiquitin–proteasome pathway, which is crucial for protein degradation and regulation under stress conditions. The identification of higher molecular mass bands suggests the presence of ubiquitinated proteins, indicating that the ECPUB5 gene product actively participates in the ubiquitination process, which is essential for protein quality control and stress response. These findings align with the known roles of polyubiquitin in tagging damaged or misfolded proteins for degradation [71,72]. Studies by Callis [73] and Moon et al. [74] have also shown that ubiquitination is a key regulatory mechanism in plants, particularly under stress conditions.

### 4.5. Future Research Perspectives

Future research should focus on the functional validation of the unique amino acid substitutions identified in ECPUB5 and their impact on the plant’s stress responses. Detailed studies examining these substitutions through site-directed mutagenesis and subsequent phenotypic analyses under various stress conditions could provide critical insights into their specific roles. Investigating the interaction of ECPUB5 with other stress-related proteins using techniques such as coimmunoprecipitation and yeast two-hybrid screens could reveal its involvement in broader stress response networks.

Additionally, exploring the expression patterns of ECPUB5 in different environmental conditions and developmental stages will help elucidate its regulatory mechanisms. This could involve transcriptomic and proteomic approaches to map the ECPUB5 expression and interaction landscapes across various biotic and abiotic stress conditions. Comparative studies with other species in the *Euphorbiaceae* family could also reveal evolutionary adaptations that confer stress tolerance, enhancing our understanding of plant resilience mechanisms. Genomic and phylogenetic analyses may uncover conserved motifs and pathways that have evolved to cope with environmental stress.

In conclusion, our study highlights the potential of ECPUB5 as a key component in the adaptive response of *E. characias* to heat and drought stress. This knowledge could be applied to improve the stress tolerance in crop species, contributing to agricultural sustainability in arid and semi-arid regions. By leveraging the insights gained from ECPUB5, future research can pave the way for the development of crops that are better equipped to withstand environmental stresses, thereby supporting food security and sustainable agriculture.

## 5. Conclusions

This study provides comprehensive insights into the molecular characteristics and seasonal expression variations of the ECPUB5 polyubiquitin gene in *E. characias*. The area where the young leaves and latex of *E. characias* were collected is characterized by annual temperatures that follow a seasonal cycle, peaking in summer (typically July–August) and reaching a low in winter (typically January–February). In contrast, precipitation is primarily concentrated in the winter months, less so in autumn and spring, and nearly absent in summer. This results in a period from approximately June to September, with no precipitation and very high temperatures, creating severe water stress conditions that require plants to adapt to survive.

Our findings underscore the potential role of the ECPUB5 gene in enhancing plant resilience to heat and drought stress, offering valuable knowledge for developing stress-tolerant crops. The unique amino acid variations identified, such as I30F, Q49H, and D39S, along with the significant upregulation of ECPUB5 under heat and drought conditions, highlight its potential role in the plant’s adaptive mechanisms. These results are in line with previous studies on the role of ubiquitin in plant stress responses, further supporting the importance of ECPUB5 in maintaining protein stability and function during environmental stress. However, it is important to note that the conclusions drawn about the gene’s role in stress tolerance require more comprehensive functional studies.

Future research should focus on experimentally validating structural predictions and further exploring the functional implications of the identified amino acid substitutions. Controlled heat and drought stress experiments as well as gene knockdown or overexpression studies are necessary to conclusively determine the role of ECPUB5 in stress tolerance. Additionally, investigating the interaction of ECPUB5 with other stress-related proteins could provide deeper insights into its role in stress tolerance. Exploring the regulatory mechanisms controlling ECPUB5 expression during stress conditions could also elucidate how plants modulate their responses to environmental challenges. Comparative studies with other species in the *Euphorbiaceae* family could reveal evolutionary adaptations that confer stress tolerance, enhancing our understanding of plant resilience mechanisms.

We acknowledge the challenges associated with replicating the natural conditions of *E. characias* in laboratory or greenhouse settings. Despite these limitations, we are committed to exploring practical alternatives to enhance the robustness of our study and to providing more definitive evidence of ECPUB5’s role in plant stress response. By leveraging the insights gained from ECPUB5, future research can pave the way for the development of crops that are better equipped to withstand environmental stresses, thereby supporting food security and sustainable agriculture.

## Data Availability

The data that support the findings of this study are available from the corresponding author upon reasonable request.

## Supplementary Materials

The following supporting information can be downloaded at: https://www.mdpi.com/article/10.3390/genes15070957/s1, Table S1. Oligonucleotides used in CODEHOP, cDNA/RNA blot, and RACE experiments. Figure S1. Multiple alignment of polyubiquitin amino acid sequences from five different plant sources. Figure S2. Alignment of ECPUB5 with polyubiquitins, consisting of five tandem repeats from other *Euphorbiaceae*.
