# Supplementary material for: ECPUB5 Polyubiquitin Gene in Euphorbia characias: Molecular Characterization and Seasonal Expression Analysis
Source: Genes (Basel). 2024 Jul 21;15(7):957. doi: 10.3390/genes15070957 (PMC11275293; doi:10.3390/genes15070957)
Supplement: Supplementary file 1 [file genes-15-00957-s001.zip › Cannea et al._Genes 2024_Figure Legends.pdf]

Supplementary Materials

Table S1. Oligonucleotides used in CODEHOP, cDNA/RNA blot, and RACE experiments.

Figure S1. Multiple alignment of polyubiquitin amino acid sequences from five different plant sources. The sequences were chosen from the GenBank SwissProt database and aligned using Clustal Omega (https://www.ebi.ac.uk/Tools/msa/clustalo/). The figure shows the alignments on which the CODEHOP primers were designed. An asterisk (*) denotes identical residues; double dots (:) represent conserved residue substitutions; a single dot (.) indicates partial conservation of the residue. The arrows above the amino acid sequences indicate the positions of the sense (F→) and antisense (←R) primers chosen from a group of candidate primers obtained from the CODEHOP program. The amino acid sequences chosen by the CODEHOP program for primer design are in bold within the boxes.

Figure S2. Alignment of ECPUB5 with polyubiquitins, consisting of five tandem repeats from other Euphorbiaceae. The amino acids of ECPUB5 that differ from those in the same positions in other proteins are highlighted within boxes. The fourth block, where no differences are observed between ECPUB5 and the other polyubiquitins, is highlighted in bold. The accession numbers of the polyubiquitins considered for alignment with ECPUB5 are as follows: WCJ41459.1 (*E. peplus*), XP_021665701.2 (*H. brasiliensis*), XP_021618731.1 (*M. esculenta*), XP_048234881.1 (*R. communis*). The numbering refers to the amino acid coding sequence.
