# Supplementary material for: ECPUB5 Polyubiquitin Gene in Euphorbia characias: Molecular Characterization and Seasonal Expression Analysis
Source: Genes (Basel). 2024 Jul 21;15(7):957. doi: 10.3390/genes15070957 (PMC11275293; doi:10.3390/genes15070957)
Supplement: Supplementary file 1 [file genes-15-00957-s001.zip › Cannea et. al._Genes 2024_Table S1.pdf]

Table S1. Oligonucleotides used in CODEHOP, cDNA/RNA blot, and RACE experiments.

Method	Sequences	Comments
CODEHOP	5'- ATGCAGATCttygtnaarac-3′	Sense primer F1 designed on the polyubiquitin conserved peptide MQIFVKTLT
	5′- TGAAGGCCAAGATCCAGgayaargargg-3′	Sense primer F2 designed on the polyubiquitin conserved peptide KAKIQDKEG
CODEHOP	5'- GGTCAGGGTCTTCACGAAGatytgcatncc - 3′	Antisense primer R1 designed on the polyubiquitin conserved peptide GMQIFVKTLT
CODEHOP	5′- TGGTCAGGAGGGATGccytcyttrtc-3′	Antisense primer R2 designed on the polyubiquitin conserved peptide DKEGIPPDQ
RACE	5′-TCTGGTTCTCCGGCTTCGTGGTGGTA-3′	SP1 antisense specific primer
RACE	5′- ACCATCTTCCAGATGTTTGCCCGCAAA-3′	SP2 antisense specific primer
RACE	5′- GACACCATTGATAATGTGAAGGCGAAG-3′	SP3 se3nse specific primer
RACE	5′-GACCACGCGTATCGATGTCGAC-TTTTTTTTTTTTTTTT-3′	Oligo (dT)-anchor primer
RACE	5′-GACCACGCGTATCGATGTCG-3′	PCR anchor primer
cDNA/RNA blot	5′-GAT AAC GTG AAA GCG AAA-3′	SP4 sense specific primer
cDNA/RNA blot	5′-AAAGATAAGCCTTTGTTGACT-3′	SP5 antisense specific primer
qRT-PCR	5′-TAGATGAATTGTTCTTGTGT-3′	SP6 sense specific primer
qRT-PCR	5′-TGGAACACAGAACAGAACCA-3′	SP7 antisense specific primer
