# Supplementary figures and images for: ECPUB5 Polyubiquitin Gene in Euphorbia characias: Molecular Characterization and Seasonal Expression Analysis
Source: Genes (Basel). 2024 Jul 21;15(7):957. doi: 10.3390/genes15070957 (PMC11275293; doi:10.3390/genes15070957)

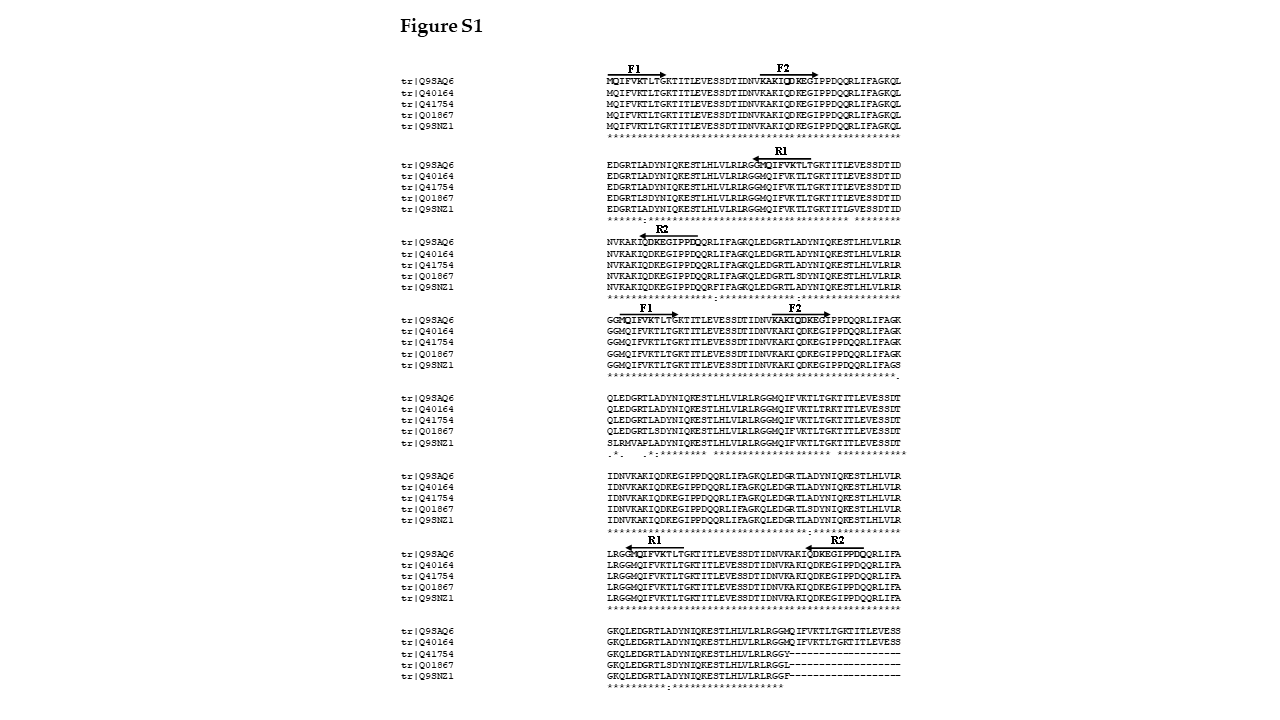

Supplement: Supplementary file 1 [file genes-15-00957-s001.zip › Cannea et al._Genes 2024_Figure S1.tif]

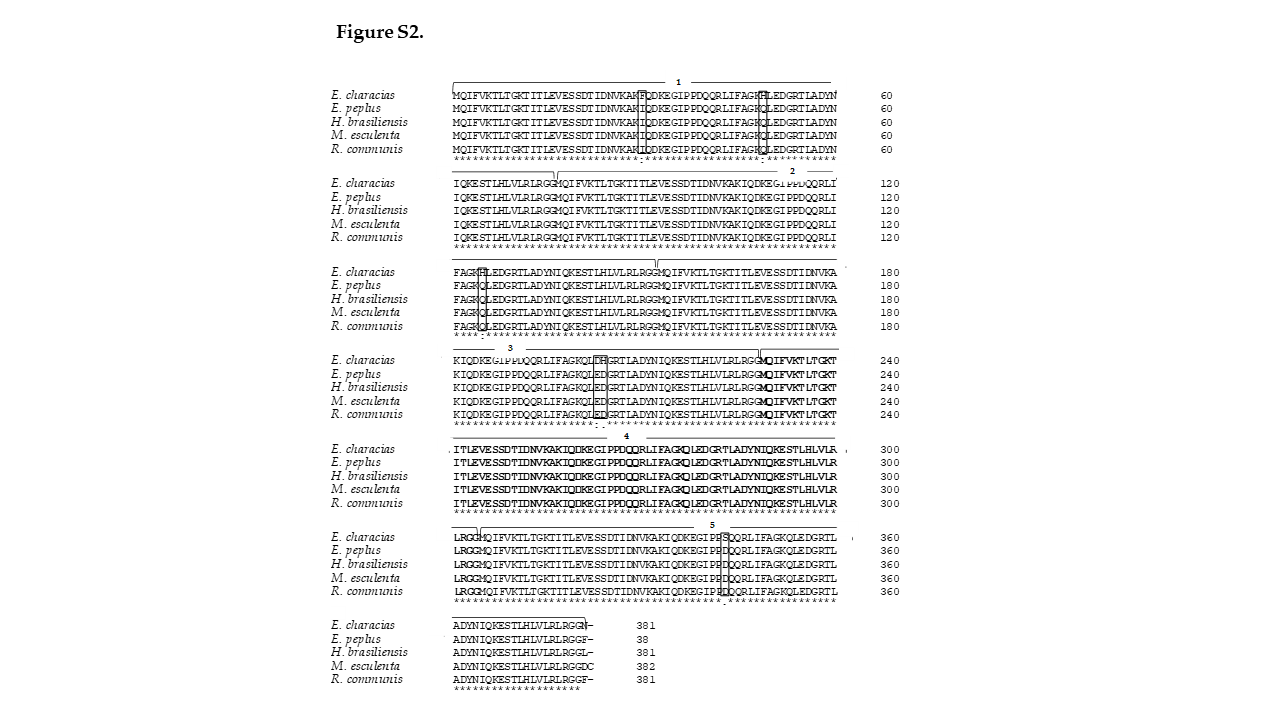

Supplement: Supplementary file 1 [file genes-15-00957-s001.zip › Cannea et al._Genes 2024_Figure S2.tif]
